# Insight into Neutral and Disease-Associated Human Genetic Variants through Interpretable Predictors

**DOI:** 10.1371/journal.pone.0120729

**Published:** 2015-03-31

**Authors:** Bastiaan A. van den Berg, Marcel J. T. Reinders, Dick de Ridder, Tjaart A. P. de Beer

**Affiliations:** 1 Delft Bioinformatics Lab, Department of Intelligent Systems, Faculty Electrical Engineering, Mathematics and Computer Science, Delft University of Technology, Mekelweg 4, 2628CD, Delft, The Netherlands; 2 European Bioinformatics Institute (EMBL-EBI) European Molecular Biology Laboratory, Wellcome Trust Genome Campus, Hinxton, Cambridge, CB10 1SD, United Kingdom; 3 Biozentrum, University of Basel, Basel 4056, Switzerland; 4 SIB Swiss Institute of Bioinformatics, Basel 4056, Switzerland; 5 Bioinformatics Group, Wageningen University, Droevendaalsesteeg 1, 6708PB, Wageningen, The Netherlands; 6 Netherlands Bioinformatics Centre, Nijmegen, The Netherlands; 7 Kluyver Centre for Genomics of Industrial Fermentation, Delft, The Netherlands; National Center for Biotechnology Information, UNITED STATES

## Abstract

A variety of methods that predict human nonsynonymous single nucleotide polymorphisms (SNPs) to be neutral or disease-associated have been developed over the last decade. These methods are used for pinpointing disease-associated variants in the many variants obtained with next-generation sequencing technologies. The high performances of current sequence-based predictors indicate that sequence data contains valuable information about a variant being neutral or disease-associated. However, most predictors do not readily disclose this information, and so it remains unclear what sequence properties are most important. Here, we show how we can obtain insight into sequence characteristics of variants and their surroundings by interpreting predictors. We used an extensive range of features derived from the variant itself, its surrounding sequence, sequence conservation, and sequence annotation, and employed linear support vector machine classifiers to enable extracting feature importance from trained predictors. Our approach is useful for providing additional information about what features are most important for the predictions made. Furthermore, for large sets of known variants, it can provide insight into the mechanisms responsible for variants being disease-associated.

## Introduction

Over the last decade, many predictors have been developed to categorize human nonsynonymous SNPs as disease-associated or neutral [1–16]. Such predictors can be used for identifying the relatively few disease-associated variants in human variation data, a type of data that is rapidly increasing due to the advances in whole genome sequencing techniques [17]. These methods typically employ large sets of known neutral and disease-associated variants to learn how to separate both classes based on variant characteristics, i.e. features. As might be expected, the degree of sequence conservation is highly predictive for disease association of genetic variants. Therefore, all available prediction methods heavily rely on conservation-based features. In fact, several methods, among which the often used method SIFT, predict class labels by thresholding a single conservation-based feature.

A comparative study, however, showed improved prediction results for methods that incorporate additional sequence-derived features [18]. It found two methods, MutPred [8] and SNPs&GO [7] to be most reliable. MutPred builds upon the SIFT score by incorporating gain and loss of structural and functional properties; SNPs&GO calculates several conservation-based features and additionally incorporates features that capture the amino acid substitution, its surrounding sequence, and features based on the functional annotation of the protein in which the substitution occurs. Except for the functional annotation-based features, these and some supplementary features were also used in this work. The more recently developed method CADD, which can be applied to all types of genetic variants, provided good performance by incorporating conservation metrics, regulatory information, transcript information, and protein level scores that are generated with methods like SIFT and PolyPhen [19].

Protein structure-based features are also attractive to further improve classification performance. However, their use is hampered by the limited availability of structural data. Furthermore, regarding the variants that do have available structure data, the fact that relatively many of these variants are disease-associated complicates the use of this type of feature by introducing a strong bias.

The fact that good classification performances can be obtained implies that the used features, which are mostly derived from sequence data, comprise valuable information about the probability of a genetic variant being neutral or disease-associated. However, this information is rarely utilized to provide better insight into what features actually contribute most to classification outcomes, i.e. what sequence characteristics are predictive for the effect of genetic variants. In this work, we show how we can obtain insight in characteristics of variations associated with disease through predictor interpretation.

**Fig 1:**
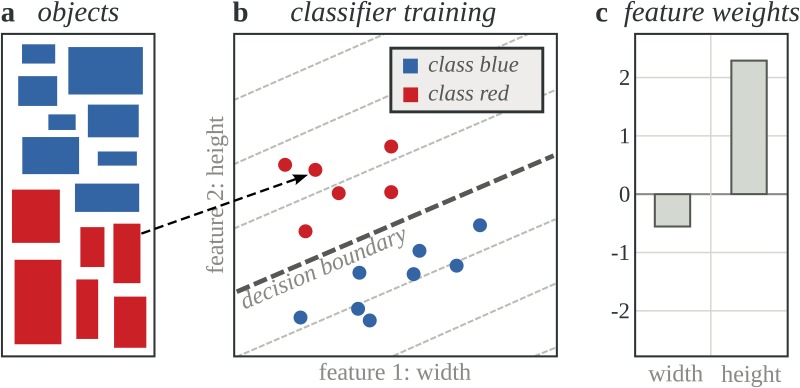
Extracting feature weights from trained classifiers. *a)* For illustration, objects in two classes (blue and red) are represented by rectangles and characterized by the features “width” and “height”. *b)* By measuring widths and heights, objects are mapped to a two dimensional grid (feature space). Classifier training results in the decision boundary that separates the two classes of objects. *c)* Feature importance can be deducted from the slope of the decision boundary. The height is more important than the width, hence the higher (absolute) weight for this feature. The sign indicates for what class the feature is predictive. Blue rectangles are generally wider, hence the negative weight for the width feature. Red rectangles are generally taller, hence the positive weight for the height feature.

We used linear support vector machines, allowing us to extract feature weights from trained classifiers. A high weight indicates a strong contribution of a certain feature to the classifier outcome, and its sign indicates if it is predictive for neutral (negative weight) or disease-associated (positive weight) variants (Fig. 1). To further enhance interpretation potential and performance of the linear classifiers, we trained separate classifiers on subsets that contain variants with the same reference amino acid. This was done based on the assumption that feature importance might be different per type of amino acid substitution. For example, a surrounding sequence with many small amino acids might be a high risk in case of substitutions from small to large amino acids, whereas substitutions from small to other small amino acids in the same surrounding might have a lower risk. Extracting feature importance from classifiers trained on the variant subsets could help in revealing such differences. Although it is not the aim of this paper to introduce a competitive predictor, we demonstrate that classifiers can be made interpretable without significant loss in prediction performance.

## Results and Discussion

**Fig 2:**
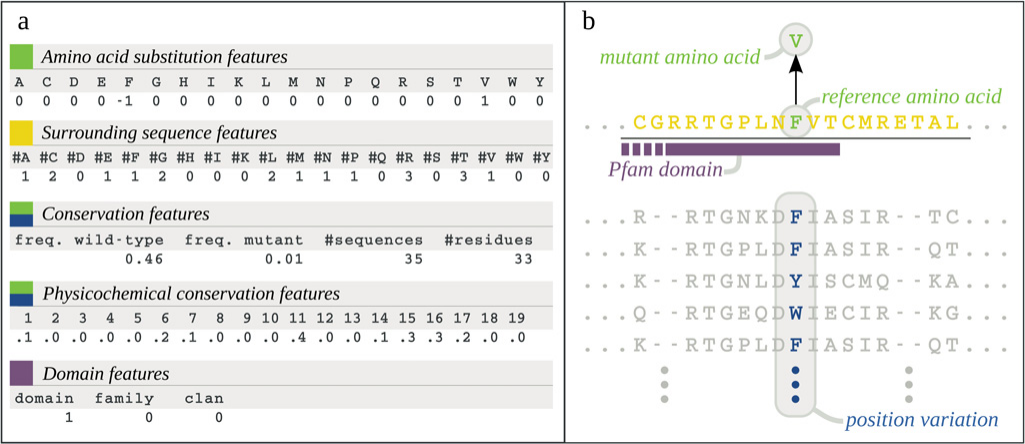
Feature categories. *a)* Five feature categories and their corresponding features. The colors indicates from which type of sequence data in part B the features were derived. *b)* Sequence and annotation data used to derive variant features; the amino acid substitution (green), the surrounding sequence (yellow), the amino acid variation in similar proteins (blue), and Pfam annotations (purple).

To characterize variants, we used five different sequence-derived feature categories (Fig. 2a) that were derived from different types of sequence data (Fig. 2b). Most of these features were inspired by the well performing method SNPs&GO [7]. The *Amino acid substitution category* consists of 20 features that capture the amino acid substitution by setting the reference amino acid to minus one, the mutant amino acid to one, and all other amino acids to zero [7]. These features were added because it is expected that different amino acid substitutions have different probabilities of resulting in a functional effect. *Surrounding sequence features* capture amino acid counts in a window of 19 residues around the substituted amino acid [7], which can be informative for structural surroundings. For example, the features could (implicitly) capture information about backbone disorder, solvent accessibility, and secondary structure. *Conservation features* capture how conserved the mutated position is based on a multiple sequence alignment (MSA) with similar proteins. Two features capture how often the reference and the mutant amino acid occur in the set of amino acids at the mutation position in the MSA (Fig. 2b). An often occurring reference amino acid indicates strong conservation and therefore a high risk of a functional effect upon mutation. In contrast, a low risk is expected in case of a high occurrence of the mutant amino acid. Two additional features capture the number of proteins in the alignment. These features were added to account for limited availability of homologous sequences, in which case the first two conservation features are expected to be less informative. *Physicochemical conservation features* capture if physicochemical properties of the mutant amino acid are much different compared to those of the amino acids at the mutation position in the MSA. These features were added based on the assumption that, for example, introducing a hydrophobic amino acid at a position where none of the amino acids at that position in the MSA is hydrophobic, might affect protein function. Finally, based on recent work showing an enrichment of deleterious variants in Pfam domains [20], *domain features* capture if a variant resides within a Pfam domain, family, or clan.

For classifier training, we used a set of 171,257 human nonsynonymous SNPs: 149,850 neutral variants from the 1000 Genomes Project and 21,407 disease-associated variants from the SwissProt *humsavar* data base [21, 22] (S1 Information). The variants were split into subsets containing variants with the same reference amino acid. Because the tryptophan, tyrosine, and phenylalanine subsets were too small for classifier training, these were combined into one subset. The resulting variant subsets are listed in Table 1. Classifiers were trained on the subsets separately. Afterwards, feature weights were extracted from the trained classifiers (Fig. 1). This was done using each of the five feature categories separately (Fig. 2a) and once using all features.

**Table 1:** Number of variants and proteins per subset.

**subset**		**# variants**	**(%)**	**# disease**	**# neutral**	**# proteins**
Alanine	A	14,852	(0.09)	1,294	13,558	7,891
Arginine	R	28,544	(0.17)	3,687	24,857	10,364
Asparagine	N	5,968	(0.03)	624	5,344	4,132
Aspartic acid	D	7,715	(0.05)	1,040	6,675	4,864
Cysteine	C	3,285	(0.02)	1,174	2,111	2,166
Glutamic acid	E	8,618	(0.05)	903	7,715	5,269
Glutamine	Q	4,723	(0.03)	435	4,288	3,467
Glycine	G	12,008	(0.07)	2,648	9,360	6,377
Histidine	H	4,319	(0.03)	532	3,787	3,170
Isoleucine	I	7,985	(0.05)	701	7,284	5,052
Leucine	L	8,206	(0.05)	1,584	6,622	4,988
Lysine	K	5,419	(0.03)	441	4,978	3,793
Methionine	M	4,950	(0.03)	503	4,447	3,598
Proline	P	11,910	(0.07)	1,152	10,758	6,587
Serine	S	11,541	(0.07)	1,165	10,376	6,522
Threonine	T	11,007	(0.06)	891	10,116	6,388
Valine	V	12,771	(0.07)	940	11,831	7,129
WYF		7,436	(0.04)	1,693	5,743	4,593
		171,257	(1.00)	21,407	149,850	16,523

For clarity, practical application of our predictor is different compared to existing methods. In our case there are 18 different classifiers instead of one. Which classifier is applied depends on the variant for which a prediction is desired. For example, if this variant results in an amino acid substitution from Glutamine (reference) to Histidine (mutant), than the classifier that is trained on all variants with reference amino acid Glutamine will be used for prediction.

### Enhanced classifier interpretation

#### Amino acid substitution features

**Fig 3:**
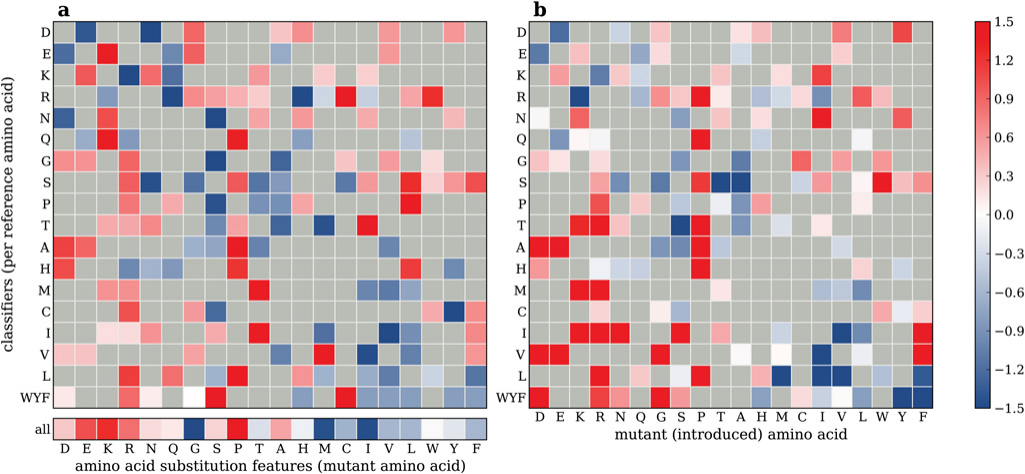
Amino acid substitution feature weights. *a)* Heat map showing feature weights obtained from classifiers trained using the amino acid substitution features. The rows show feature weights obtained per variant subset classifier. The single row at the bottom shows feature weights obtained from a classifier trained on the entire set of variants. The rows and columns are ordered based on amino acid properties [23]. Low (blue) and high (red) weights indicate that the feature is predictive for neutral and disease-associated variants respectively. Gray cells indicate amino acid substitutions that do not occur in the data set, because these substitutions require more than one mutation in the reference codon. *b)* Heat map showing log odds ratios between neutral and disease-associated variants that were obtained by counting the amino acid substitutions in our data set. Here, low (blue) and high (red) values indicate that substitutions occur relatively often in the set of neutral and disease-associated variants, respectively.

Extracted feature weights from classifiers trained using the *amino acid substitution features* are visualized using a heat map in Fig. 3a. Here, each row shows feature weights obtained from one subset classifier, i.e. a classifier trained on one of the variant subsets. For example, the colors in the top row correspond to the weights obtained from the classifier trained on all variants with aspartic acid (D) as reference amino acid. A positive weight (red) indicates that the feature (the mutant amino acid in this case) is predictive for disease-association whereas a negative weight (blue) indicates importance for neutral variants. The higher the (absolute) weight, the higher the feature importance. Using the top row as example again, the low weight of the glutamic acid feature (column E) indicates that a substitution from aspartic acid to glutamic acid is relatively safe, whereas the high weight of the glycine feature (column G) indicates that a substitution from aspartic acid to glycine is relatively dangerous. Gray elements indicate amino acid substitutions that do not occur in our data set, since these require more than one mutation at the nucleotide level. Additionally, the feature weights obtained from the classifier that was trained on the entire data set are shown in the single row at the bottom.

The classifier trained on the entire data set (bottom row) only has twenty features to capture the risks of the different amino acid substitutions. For interpretation, the low weight of the methionine feature indicates that substitutions from and to methionine are relatively safe. In contrast, the weights of the subset classifiers offer much richer interpretations. Here it can be observed that substitutions from threonine to methionine are relatively safe, but that substitutions the other way around (from methionine to threonine) are relatively dangerous.

For validation, the heat map in Fig. 3b shows the log odds ratios between the neutral and disease-associated variants in our data set that were calculated using the amino acid substitution counts. Here, high values indicate relatively dangerous variants, i.e. variants that are relatively often disease related, and low values indicate relatively safe variants. The feature weights of the subset classifiers in Fig. 3a clearly reflect the log odds ratios, thereby showing that the subset classifiers successfully learned the ‘risks’ of the different amino acid substitutions.

#### Surrounding sequence features

**Fig 4:**
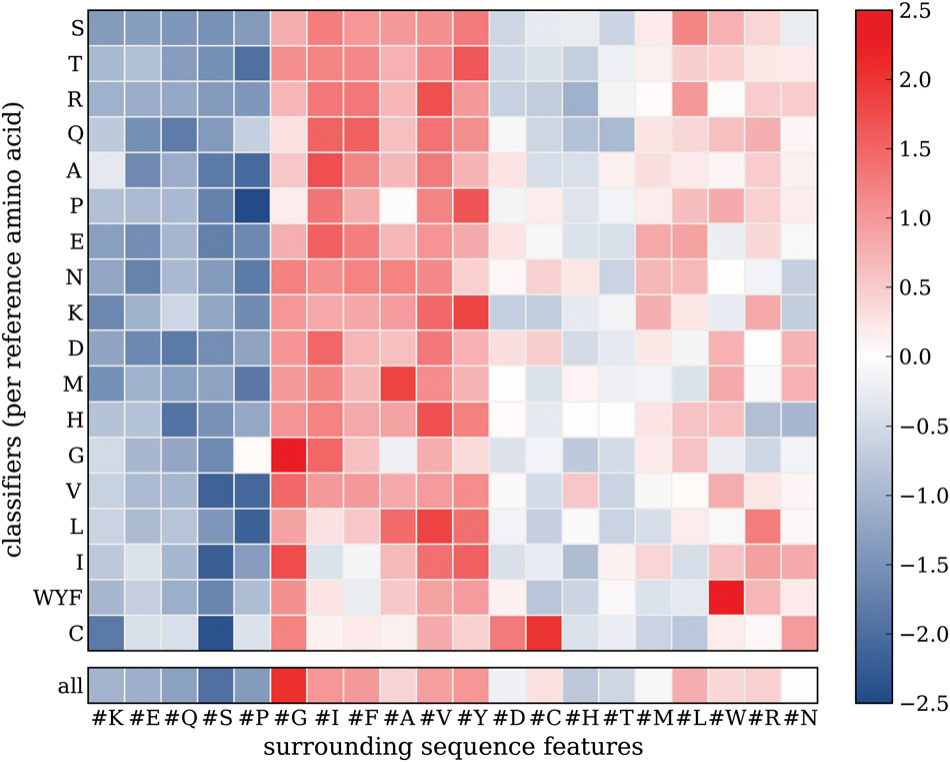
Surrounding sequence feature weights. Heat maps showing feature weights obtained from classifiers trained using the surrounding sequence features. The rows show feature weights obtained per variant subset classifier. Both the rows and the columns are hierarchically clustered (complete linkage). The single row at the bottom shows feature weights obtained from a classifier trained on the entire set of variants. Low (blue) and high (red) weights indicate that the feature is predictive for neutral and disease-associated variants respectively.

Resulting weights of classifiers trained using the *surrounding sequence features* are shown in Fig. 4. In this case, most columns show consistently signed weights, which indicates that the same general rules hold for different amino acid substitutions. For example, it is easy to observe that in a serine-rich surrounding (#S), any amino acid substitution is relatively safe, independent of what the reference amino acid is. The weights of the classifier trained on the entire set of variants (bottom row) show that the same rules can indeed be learned using the entire set of variants.

For the sequence surrounding features, enhancing interpretation by using the subset approach therefore seems limited. However, some interesting details can still be observed that cannot be derived from the classifier trained on the entire data set. For example, the cysteine subset classifier (C) shows a very negative weight for the cysteine count feature (#C), indicating that in a cysteine-rich surrounding, substituting cysteines is relatively dangerous. This might be explained by the fact that such variants potentially break disulfide bridges [24]. Similarly, in a glycine-rich surrounding (#G), substituting glycines (G) shows a relatively high risk of being disease-associated, which might be related to changing conformational entropy of a flexible region.

The columns were clustered using hierarchical clustering (complete linkage), which reveals a cluster with positive values (red cluster) containing hydrophobic amino acids. This indicates that amino acid substitutions are relatively dangerous in a hydrophobic sequence surrounding, which is consistent with the fact that variants in the hydrophobic protein core have a high-risk of disrupting thermodynamic stability.

#### Physicochemical conservation features

The *physicochemical conservation features* capture whether there is a large physicochemical distance between the mutant amino acid and the amino acids at the same position in the MSA (Fig. 2b). For defining physicochemical distances, we used so called amino acid scales: mappings from amino acids to corresponding values that capture some physicochemical property, e.g. hydrophobicity. Many amino acid scales are collected in the AAIndex database [25], but the majority of these scales are highly correlated. We therefore used 19 amino acid scales that were derived from the AAIndex database using VARIMAX [26]. This set contains independent amino acid scales (which is desired for classification performance) of which as many as possible are still closely related to physicochemical properties (which is desired for interpretation). The amino acid scales that have a strong correlation to physicochemical properties, i.e. the interpretable scales, are given in Table 2. The AAIndex scales that best correlate to the derived scales are given in S1 Table.

**Table 2:** The amino acid scales that corresponds the best to physicochemical properties.

**Scale**	**Property**
1	Hydrophobicity, *β*-sheet
2	*α*-Helix
3	Bulkiness (volume/size/mass)
4	Amino acid composition
7	Isoelectric point
8	*β*-sheet

For calculating these features, the amino acids are first mapped to characterizing values using the amino acid scale, after which the minimal distance between the mutant amino acid and the amino acids at the same position in the MSA is calculated. This is done for all 19 amino acid scales. As an example, a large mutant amino acid on a position where the MSA contains only small amino acids will result in a large bulkiness (scale 3) distance. These features basically capture conservation of physicochemical properties.

**Fig 5:**
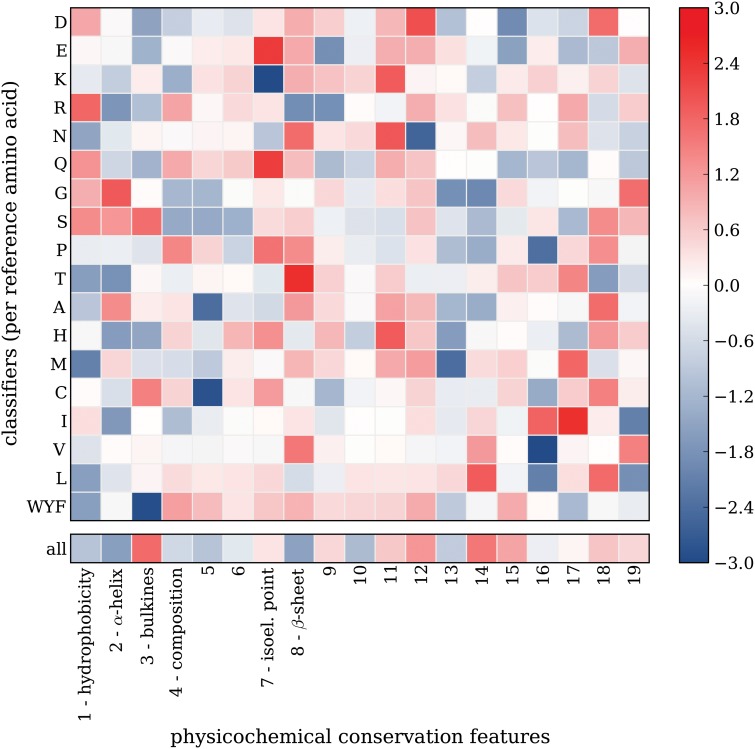
Physicochemical conservation feature weights. Heat maps showing feature weights obtained from classifiers trained using the physicochemical conservation features. The rows show feature weights obtained per subset classifier. The single row at the bottom shows feature weights obtained from a classifier trained on the entire set of variants. Low (blue) and high (red) weights indicate that the feature is predictive for neutral and disease-associated variants respectively.

This category captures a property of the mutant amino acid, while our classifiers are trained on variants with the same reference amino acid, which complicates interpretation. Theoretically, splitting the variants per amino acid substitution (150 out of the 380 possible substitutions, since we only consider substitutions that are a result of a single mutation in the codon) could improve interpretation possibilities, but these subsets would be too small for classifier training. Still, some intuitive results can be observed (Fig. 5). For example, cysteines are small and often buried, so replacing these by a large amino acid may disrupt protein core packing. Conversely, a difference in bulkiness when replacing the relatively large amino acids phenylalanine, tyrosine or tryptophan, is found to be relatively safe.

#### Conservation and domain features

The *conservation features* indicate how conserved a mutated position is. As expected, variants for which the reference amino acid often occur at the same position in homologous sequences have a high risk of being disease-associated, and variants for which the mutant amino acid often occur on the same position in homologous sequences are relatively safe (S1 Fig.). These rules hold for all variants, independent of what amino acid substitution they induce. Similarly, considering the *domain features*, it holds for all variants that the risk of being disease-associated is relatively high if it resides in a Pfam domain (S2 Fig.). For these features, the classifiers trained on the variant subsets therefore do not provide better interpretations than the classifier trained on all variants.

#### All features combined

Considering the classifiers trained using *all features*, the resulting feature weights (S3 Fig.) show that the conservation and domain features generally obtain high (absolute) weights, indicating that these feature categories are most predictive. However, high weights for certain features in other categories show that these also contribute to prediction and interpretation. For example, for variants resulting in alanine substitutions, not only high conservation is a strong indicator for disease-association, but also if the alanine is substituted to an aspartic (or glutamic) acid. For the set of variants with phenylalanine, tryptophan, and tyrosine as reference amino acid, it can be observed that substitutions to less bulky amino acids, and especially to cysteines, have a relatively low risk of being disease associated.

### Classifier performances

Interpreting a classifier is only useful if it demonstrates good prediction performance, as otherwise the used features are not predictive and consequently interpretation of their weights is dangerous. To assess classifier performance, we used ten-fold cross-validation using the area under the receiver operator curve (AUC) as performance measure. Again, classifiers were tested using each feature category separately and one classifier was tested using all features. Classifiers were tested for all variant subsets; to obtain a combined subset classifier (*C*_*S*_) result, all test set predictions were combined to generate an ROC-curve. For comparison, classifiers that were trained on the entire set of variants (*C*_*E*_) were also tested.

**Fig 6:**
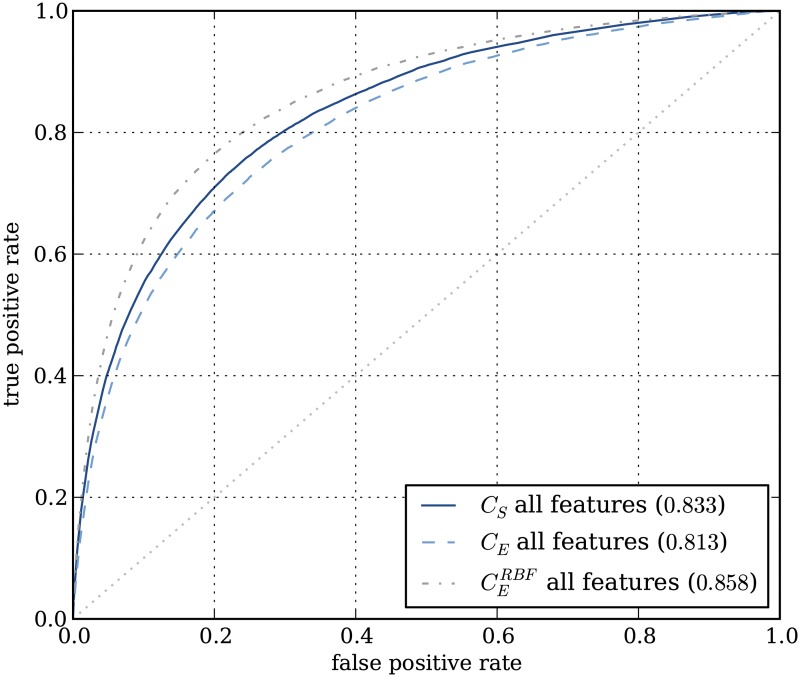
ROC-curves showing classifier performances using all features. In blue, performances for linear support machines using the combined subset classifier approach (*C*_*S*_), and for a classifier trained on the entire set of variants (*C*_*E*_). In gray the performance of a non-linear support vector machine (RBF kernel) trained on the entire set of variants.

Resulting performances are given in Table 3 (more results can be found in S2 Table and S4 Fig.). In case of the linear support vector machines, subset classifiers (*C*_*S*_) consistently outperformed the classifiers trained on the entire set of variations (*C*_*E*_). The subset approach thus not only improves interpretation, it also results in better classification performances (for linear classifiers). Best performance was obtained using the subset classifier trained on all features, resulting in 0.833 AUC (Fig. 6).

**Table 3:** Classification performances (AUC).

**Features**	*C_S_* *	*C_E_* **	Δ
*Linear support vector machine classifiers*			
all features	0.833	0.813	0.020
amino acid substitution	0.683	0.587	0.096
surrounding sequence	0.714	0.673	0.041
conservation	0.775	0.765	0.010
physicochemical conservation	0.712	0.633	0.079
domain	0.720	0.676	0.044
*RBF support vector machine classifiers*			
all features	0.845	0.858	-0.013
*Other prediction methods*			
SIFT	-	0.803	-
PolyPhen 2	-	0.807	-

* combined subset classifiers,

** classifier trained on all variants

**Fig 7:**
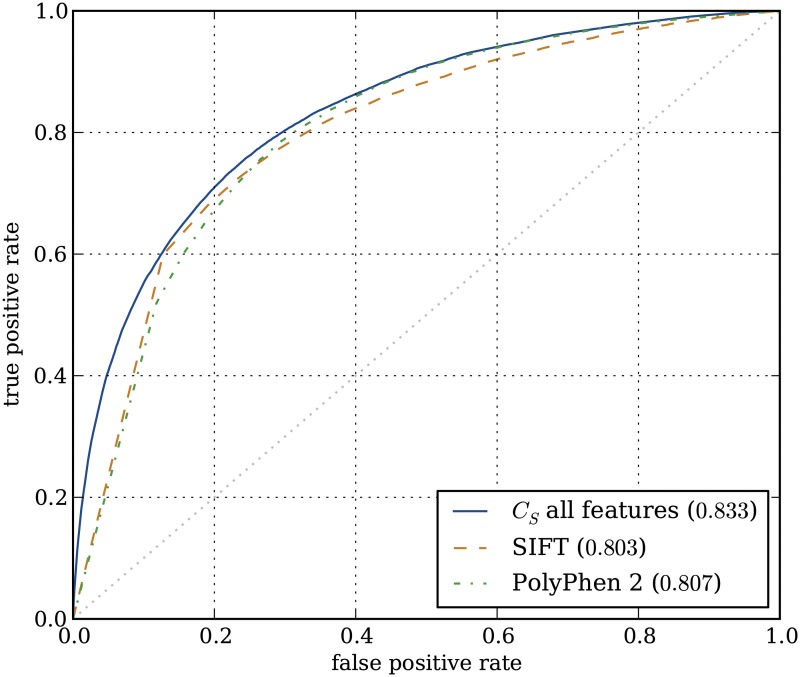
ROC-curves showing classifier performance compared to SIFT and PolyPhen 2. In blue, performance using the combined subset classifier approach (*C*_*S*_). In orange and green, performances of SIFT and Polyphen 2 respectively.

To compare this result with existing methods, we applied the two often used methods SIFT and PolyPhen2 to our data set. With AUCs of 0.803 and 0.807 respectively, both methods were outperformed by our interpretable classifier (Fig. 7). We also compared our linear approach to one using a non-linear classifier, which may be better suited for a complex classification problem such as this. With a cross-validation result of 0.858 (Fig. 6), this was indeed the case for a non-linear SVM (RBF kernel). However, this classifier does not allow for interpretation. By using the subset approach with linear classifiers, we managed to enable interpretation with only a limited loss in performance (0.833 vs. 0.858).

## Conclusion

In this work, we propose to investigate properties of disease-causing genetic variants, by exploiting predictors trained to distinguish between such variants and neutral mutations. We take a linear classification approach, allowing us to interpret feature weights in a straightforward manner. The results showed that our approach enables interpretation with only limited performance loss compared to the use of non-linear classifiers. This is useful for users that are interested in specific disease-associated variants, providing better understanding about mechanisms potentially responsible for functional effects. Furthermore, when considering large sets of variants, the approach also provides pointers to help find general mechanisms resulting in neutral or disease-associated variants.

## Methods

Human protein sequences were obtained from the UniProt website (June 3, 2013) using a query for canonical human proteins with keyword “Complete proteome”, and from the Ensembl (version 71) FTP server (see S1 Information for the URLs used). Only the protein sequences that were identical in both sets were selected, thereby providing a one-to-one mapping from UniProt to Ensembl proteins (S4 File), which facilitated running other prediction methods on our data. Proteins longer than 10,000 residues were considered outliers and therefore removed. This resulted in a set of 18,162 human protein sequences (S3 File).

### Human variants

Disease-associated variants were obtained from the SwissProt human single amino acid variants data base (humsavar release 2013–07), selecting all variants with annotation disease. Non-disease associated variants from the 1000 Genomes Project were obtained by directly querying the database (Dec 2012). An overlap of 676 variants that were both found in the set of disease-associated variants and the set of neutral variants were assumed to be disease-associated and therefore removed from the neutral set. Synonymous SNPs, duplicate variants, variants in the start codon, and substitutions that included other than the twenty unambiguous amino acids were removed. To prevent a bias caused by an unbalanced occurrence of multiple nucleotide mutations in the different classes [22], amino acid substitutions that require more than one mutation in the codon were removed. This resulted in 23,039 disease-associated variants in 1,941 proteins and 216,697 neutral variants in 17,183 proteins.

The human protein sequences were used to filter out variants that do not “fit” the protein sequence, i.e. variants for which the reference amino acid was not found on the specified position in the sequence. Variants for which no protein sequence was available were removed. Also, variants at different locations with an identical surrounding sequence in a window of nineteen amino acids around the mutation were removed, assuming a mapping from the same DNA mutation to multiple proteins. The resulting data set consists of 171,257 variants in 16,523 proteins, subdivided into 149,850 neutral and 21,407 disease-associated variants (S1 File and S2 File).

The variants were split into twenty subsets, each containing variants with the same reference amino acid. Due to the low number of substituted tryptophans, tyrosines, and phenylalanines, these subsets were combined into one subset, resulting in a total of eighteen subsets. The number of variants per subset are given in Table 1.

### Feature categories

Calculation of the different feature categories is described below. A file with feature matrix data (250 MB) is available on request.

#### Amino acid substitution features

Amino acid substitutions are represented by twenty features, one per amino acid, in which the reference amino acid (S2 File, column 3) is set to −1, the mutant amino acid (S2 File, column 4) is set to 1, and all other amino acids are set to 0 (Fig. 2). For each variant subset, some features have the same value for each variant in that set. Therefore, these features do not contribute to the classification and were removed. For example, the serine feature was removed from the variant subset with substitutions from serine to other amino acids, because that feature is −1 for all variants in the subset. Also, the aspartic acid, glutamic acid, histidine, lysine, methionine, glutamine, and valine features were removed, because a substitution from serine to any of these amino acids requires more than one mutation in a serine codon, and such mutations are not present in our data set. These feature values are therefore all 0.

#### Surrounding sequence features

Twenty features, one per amino acid, capture the surrounding sequence of each variant (S2 File, column 6). These twenty features contain amino acid counts of a sequence window of 19 residues around the variant (Fig. 2a).

#### Conservation features

Alignments with similar proteins were obtained for each human protein by running a single HHBlits [27] against the redundancy reduced UniProt20 data base version 2013–03 using default parameter settings (S1 Information). For each variant, four conservation features were derived from the multiple sequence alignment (MSA) column at the mutation position: *i)* the frequency of occurrence of the reference amino acid, *ii)* the frequency of occurrence of the mutant amino acid, *iii)* the total number of aligned proteins, and *iv)* the number of aligned residues in this column.

#### Physicochemical conservation features

These features employ the MSA to capture minimal physicochemical distances between the mutant amino acid and the set of variant amino acids at the mutation position (Fig. 2b), in which amino acid scales were used to calculate physicochemical distances between two amino acids. Amino acid scales map each amino acid to a value that captures a physicochemical or biochemical property and the AAIndex data base [25] contains a large collection of these scales, many of which are highly correlated. We therefore used a set of 19 uncorrelated scales derived from the entire AAIndex database [26]. The uncorrelated scales were derived in such a way that some of the scales remain highly correlated to a set of consensus natural scales: Scale 1 has strong correlation with hydrophobicity and *β*-sheet scales, scale 2 has strong correlation with *α*-helix scales, scale 3 has strong correlation with bulkiness scales, scale 4 has strong correlation with amino acid composition scales, and scale 7 has strong correlation with isoelectric point scales (Fig. 5b in [26]). This way, all amino acid scales data is captured while interpretation is still possible for some of the resulting uncorrelated scales.

#### Domain features

Pfam version 27.0 [28] was used to predict Pfam domains on the protein sequences (S1 Information). Resulting annotations (S5 File) were used to construct three binary domain features that are set to 1 if the variant resides within a predicted Pfam family, domain, or clan, respectively, or to 0 otherwise.

### Classification

A linear support vector machine (LIBSVM [29]) was employed for classification [30], using a linear and RBF kernel for the linear and non-linear classifiers respectively, and using a 10-fold stratified cross-validation (CV) protocol to asses classifier performance [31]. When using the linear kernel, parameter *C* was set to 0.1; for the RBF kernel we set *C* = 1.0 and *γ* = 0.01. Probability estimates were used as classifier output, so that outcomes of the different subset classifiers could be combined.

Classifiers were trained on the variant subsets separately (*C*_*S*_). Their combined performance was obtained by combining the outcomes of all CV test sets for all subset classifiers and using these to generate an ROC-curve [32] for the entire data set. The area under the ROC-curve (AUC) was used as performance measure. Classifiers were also trained on the entire set of variants (*C*_*E*_), in which case the average AUC of the ten CV-loops was used as performance measure. For classifier types, *C*_*E*_ and *C*_*S*_, a classifier was trained for each of the feature categories, and a classifier was trained on all features. Feature scaling was applied to enable the use of data with varying ranges. All feature values were standardized (the feature value subtracted by the mean of the feature vector and the result divided by the standard deviation of the feature vector) so that all feature vectors have zero-mean and unit-variance.

After cross-validation, classifiers were trained on the entire data sets. These classifiers were used to obtain feature weights. For a given set of variants *V*, the feature weight vector **w** from the trained SVM classifier was obtained using:

$$\begin{array}{c} w=\sum_{v_{i}\in V}\alpha_{i}y_{i}\Phi \left(v_{i}\right), \end{array}$$ (1)

in which *α*_*i*_ are the weights assigned to the objects (variants), *y*_*i*_ are the variant labels (−1 for neutral and 1 for disease-associated) and Φ(*v*_*i*_) is a function that maps a variation *v*_*i*_ to its feature representation. For comparison, weight vectors are standardized to zero mean and unit standard deviation.

### Other prediction methods

Predictions for our mutation data set were obtained using the two often used prediction methods SIFT [1] and PolyPhen2 [9]. SIFT predictions were obtained using their website, the resulting SIFT scores were used as prediction outcome. Predictions were missing for a total of 4,208 mutations, either because the protein ID (ENSP) or the requested position in the sequence was not found by the current SIFT predictor. PolyPhen2 predictions were also obtained using their website. Both our list of mutations and the FASTA file with human proteins were supplied to the method, which was run using HumDiv as classifier model, GRCh37/hg19 as genome assembly, canonical transcripts, and missense annotations. The resulting Naive Bayes posterior probabilities were used as prediction outcome. No predictions were given for 647 variants. The area under the ROC-curve was used as performance measure.

## Supporting Information

S1 Information Supporting information. This file describes which web resources were used to get sequence and variant data, and it describes how the software tools HHBlits and PfamScan were used to obtain the multiple sequence alignments and the PFAM domains, respectively, that were used for feature calculation.
(PDF)

S1 File Labeled human variants. This file contains the labeled variants that were used for classifier development and testing (using cross-validation). The first line contains the two used labels: neutral (0) and disease (1). The following lines each contain two tab-separated items: a variant id, and the corresponding label (0 or 1). The variant id is composed of four underscore-separated items: the protein id (UniProt), the protein sequence position (starting at 1), the reference amino acid, and the mutant amino acid.
(TXT)

S2 File Variant data. This file contains sequence information for all variants. Each row contains twelve tab separated items related to one variant: 1. the protein id (UniProt), 2. the protein sequence position (starting at 1), 3. the reference amino acid,4. the mutant amino acid, 5. the label (0 for neutral, 1 for disease), 6. the surrounding amino acid sequence, 7. the window size of the surrounding sequence, 8. the surrounding nucleotide sequence, 9. the reference codon, 10. the mutant codon, 11. the id of the pdb structure to which the variant is mapped (None if we could not map the variant to a structure in the pdb), 12. the position in the pdb structure to which the variant is mapped (−1 in case of no mapping).
(TXT)

S3 File Protein sequences. This file contains the human protein sequences that are used in this work. The sequences are in FASTA format with UniProt identifiers.
(FSA)

S4 File UniProt to Enseble id mapping. This file contains a mapping from UniProt to Ensembl identifiers for the human proteins in S3 File.
(TXT)

S5 File Predicted PFAM domains. This file contains the raw output of running PfamScan on the set of human proteins in S3 File.
(TXT)

S1 Fig Conservation feature weights. Heat maps showing feature weights obtained from classifiers trained using the conservation features. The rows show feature weights obtained per subset classifier. The single row at the bottom shows feature weights obtained from a classifier trained on the entire set of variants. Low (blue) and high (red) weights indicate that the feature is predictive for neutral and disease-associated variants respectively.
(PDF)

S2 Fig Domain feature weights. Heat maps showing feature weights obtained from classifiers trained using the domain features. The rows show feature weights obtained per subset classifier. The single row at the bottom shows feature weights obtained from a classifier trained on the entire set of variants. Low (blue) and high (red) weights indicate that the feature is predictive for neutral and disease-associated variants respectively.
(PDF)

S3 Fig All feature weights. Heat maps showing feature weights obtained from classifiers trained using all features. The rows show feature weights obtained per subset classifier. Both the rows and the columns are hierarchically clustered (complete linkage). The single row at the bottom shows feature weights obtained from a classifier trained on the entire set of variants. Low (blue) and high (red) weights indicate that the feature is predictive for neutral and disease-associated variants respectively.
(PDF)

S4 Fig Classifier performances using all features. *a)* Classifier performances using the entire data set. *b)* Classification performances per variant subset.
(PDF)

S1 Table AAIndex scales. The AAIndex amino acid scales with highest correlation (*r*) to the varimax-derived scales (*V*) as taken from [26].
(PDF)

S2 Table Classifier performances. (PDF)
