# Supplementary material for: Insight into Neutral and Disease-Associated Human Genetic Variants through Interpretable Predictors
Source: PLoS One. 2015 Mar 31;10(3):e0120729. doi: 10.1371/journal.pone.0120729 (PMC4380319; doi:10.1371/journal.pone.0120729)
Supplement: S1 Information — This file describes which web resources were used to get sequence and variant data, and it describes how the software tools HHBlits and PfamScan were used to obtain the multiple sequence alignments and the PFAM domains, respectively, that were used for feature calculation. (PDF) [file pone.0120729.s001.pdf]

Supporting Information:

Insight into neutral and disease-associated human genetic variants through interpretable predictors

Bastiaan A. van den Berg[1,6,7], Marcel J.T. Reinders[1,6,7], Dick de Ridder[1,5,6,7], Tjaart A.P. de Beer[2,3,4,*]

1 Delft Bioinformatics Lab, Department of Intelligent Systems, Faculty Electrical Engineering, Mathematics and Computer Science, Delft University of Technology, Mekelweg 4, 2628CD, Delft, The Netherlands
2 European Bioinformatics Institute (EMBL-EBI) European Molecular Biology Laboratory, Wellcome Trust Genome Campus, Hinxton, Cambridge, CB10 1SD, United Kingdom
3 Biozentrum, University of Basel, Basel 4056, Switzerland
4 SIB Swiss Institute of Bioinformatics, Basel 4056, Switzerland
5 Bioinformatics Group, Wageningen University, Droevendaalsesteeg 1, 6708PB, Wageningen, The Netherlands
6 Netherlands Bioinformatics Centre, Nijmegen, The Netherlands
7 Kluyver Centre for Genomics of Industrial Fermentation, Delft, The Netherlands
∗ E-mail: Corresponding tjaart.debeer@unibas.ch

Used web resources

Fasta files with human protein sequences were obtained from the Ensemble FTP server[1], and from the UniProt website[2]. Human variation data was obtained from the SwissProt human single amino acid variations (humsavar) data base[3]. Only the variants annotated as disease-associated were used for our data set. Predictions for the variations in our data set were obtained using the SIFT website[4] and using the PolyPhen2 website[5].

Generating MSA's using HHBlits

A local installation of the software tool HHBlits[6,7] was used to generate MSA's for the set of human proteins in File S4. A script was used to run the following command for each protein in this set:

```
hhblits -i A0A183.fsa -o $OUTPUT/A0A183.hhr \
    -oa3m $OUTPUT/A0A183.a3m -n 1 \
    -d $DATABASES/hhsuite/uniprot20_2013_03/uniprot20_2013_03 \
    -cpu 8 -maxfilt 500000 -maxmem 50GB
```

The raw output files (`.a3m`) were parsed to generate files with the aligned sequences only, these files were used for calculating the MSA-based features. Zip files with the raw output (4.4 GB) and the generated alignment files (2.9 GB) are available on request.

[1] ftp://ftp.ensembl.org/pub/release-71/fasta/homo_sapiens/pep/Homo_sapiens.GRCh37.71.pep.all.fa.gz
[2] http://www.uniprot.org/uniprot/?query=organism:9606+AND+keyword:"Complete+proteome+(KW-0181)"+reviewed:yes&force=yes&format=fasta
[3] http://www.uniprot.org/docs/humsavar
[4] http://sift.jcvi.org/www/SIFT_enst_submit.html
[5] http://genetics.bwh.harvard.edu/pph2/bgi.shtml
[6] http://toolkit.tuebingen.mpg.de/hhblits
[7] ftp://toolkit.genzentrum.lmu.de/pub/HH-suite/

Predicting PFAM domains

A local installation of the software tool PfamScan[8] was used to predict PFAM domains for the set of human proteins in File S4, using the following command:

```
./pfam_scan.pl -fasta protein_sequences.fsa \
    -dir $HOME/tools/pfam_scan/hmm \
    -outfile pfam_raw.txt -as -cpu 1
```

The raw output of this software tool is given in File S5. A parsed version of this file was used to calculate the PFAM-based features.

[8] http://ftp.ebi.ac.uk/pub/databases/Pfam/Tools/PfamScan.tar.gz
