# Supplementary figures and images for: Insight into Neutral and Disease-Associated Human Genetic Variants through Interpretable Predictors
Source: PLoS One. 2015 Mar 31;10(3):e0120729. doi: 10.1371/journal.pone.0120729 (PMC4380319; doi:10.1371/journal.pone.0120729)

classifiers (per reference amino acid)

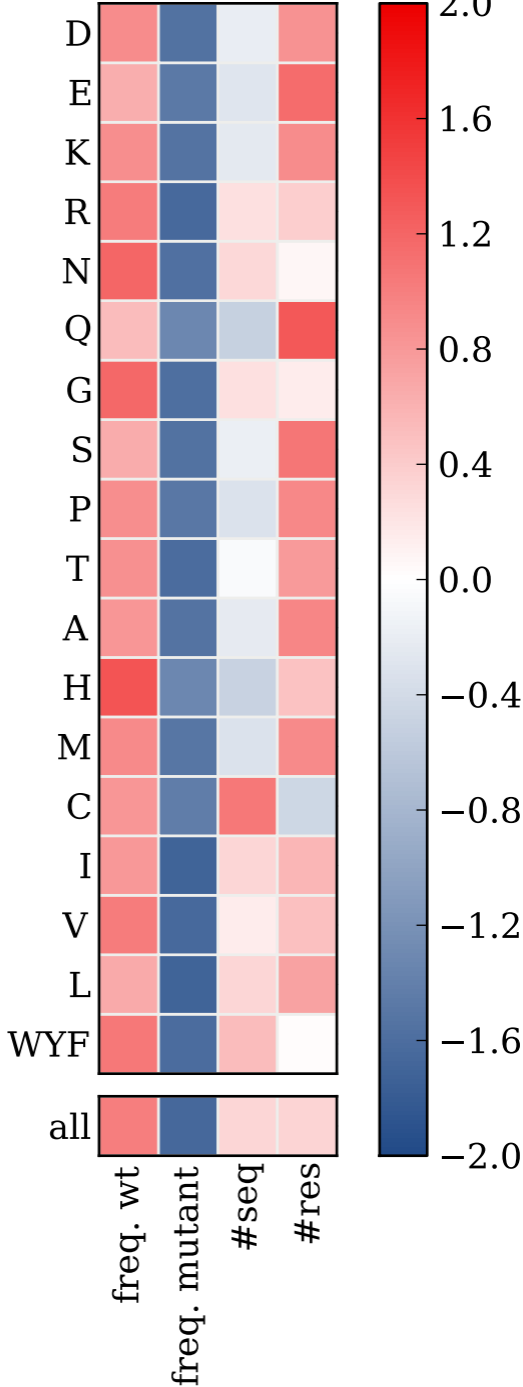

conservation features

Supplement: S1 Fig — Heat maps showing feature weights obtained from classifiers trained using the conservation features. The rows show feature weights obtained per subset classifier. The single row at the bottom shows feature weights obtained from a classifier trained on the entire set of variants. Low (blue) and high (red) weights indicate that the feature is predictive for neutral and disease-associated variants respectively. (PDF) [file pone.0120729.s007.pdf]

classifiers (per reference amino acid)

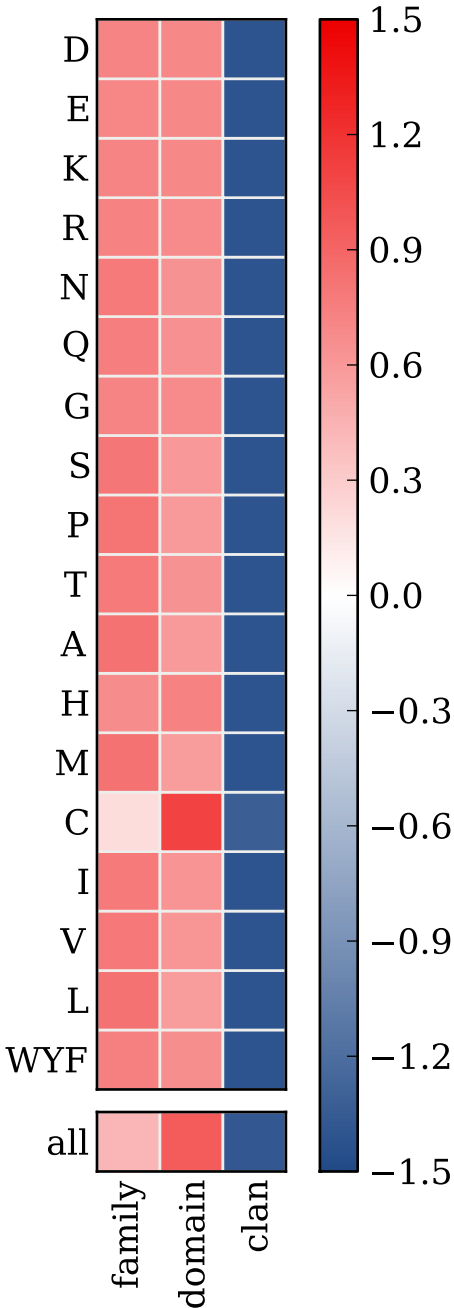

domain features

Supplement: S2 Fig — Heat maps showing feature weights obtained from classifiers trained using the domain features. The rows show feature weights obtained per subset classifier. The single row at the bottom shows feature weights obtained from a classifier trained on the entire set of variants. Low (blue) and high (red) weights indicate that the feature is predictive for neutral and disease-associated variants respectively. (PDF) [file pone.0120729.s008.pdf]

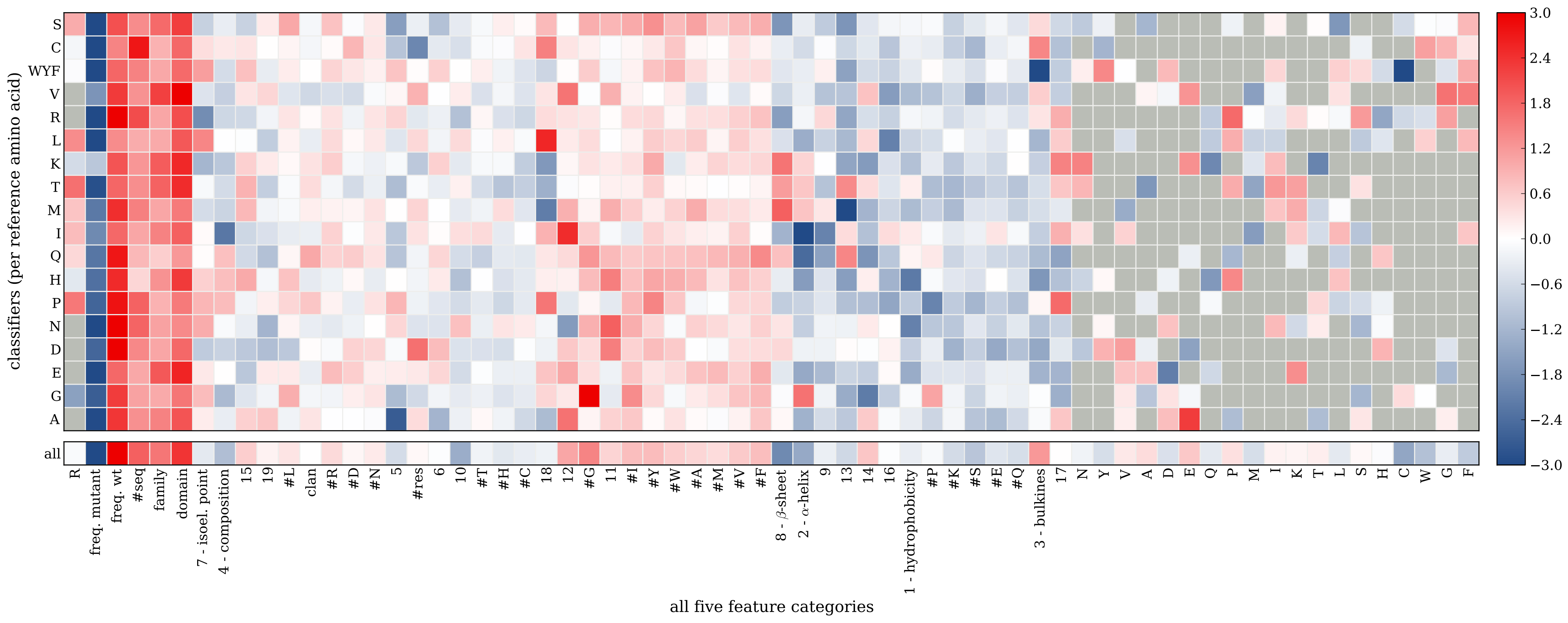

Supplement: S3 Fig — Heat maps showing feature weights obtained from classifiers trained using all features. The rows show feature weights obtained per subset classifier. Both the rows and the columns are hierarchically clustered (complete linkage). The single row at the bottom shows feature weights obtained from a classifier trained on the entire set of variants. Low (blue) and high (red) weights indicate that the feature is predictive for neutral and disease-associated variants respectively. (PDF) [file pone.0120729.s009.pdf]

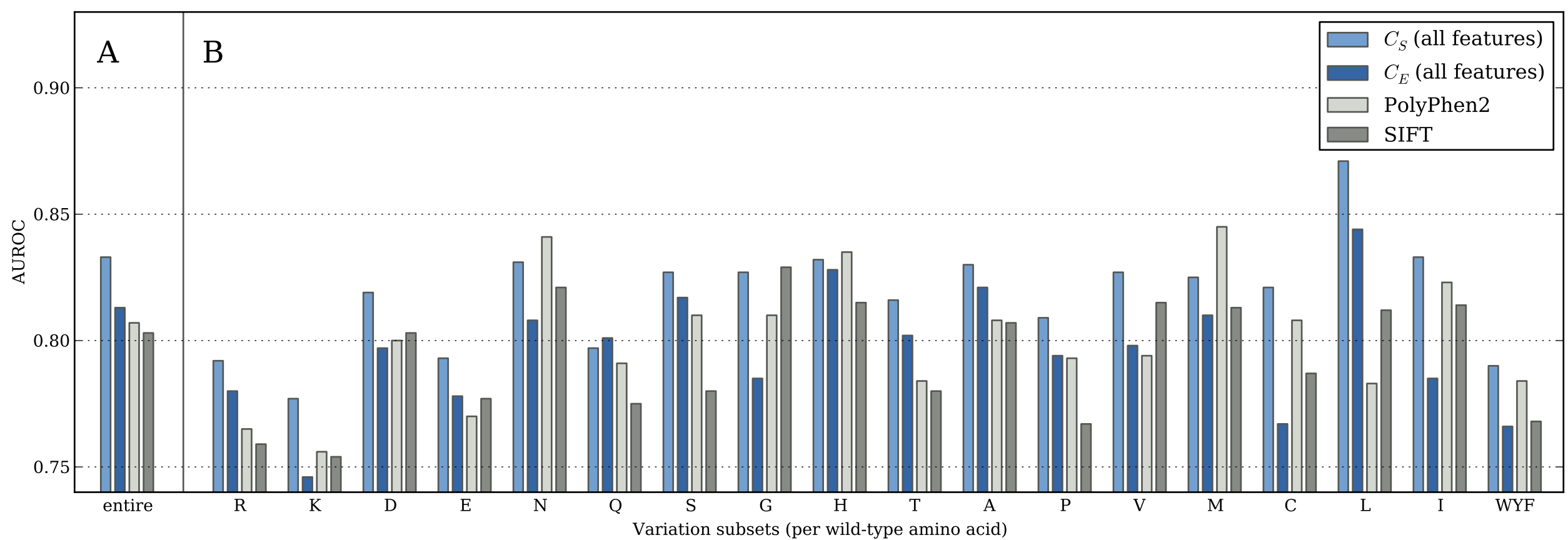

Supplement: S4 Fig — a) Classifier performances using the entire data set. b) Classification performances per variant subset. (PDF) [file pone.0120729.s010.pdf]
