# Supplementary material for: Insight into Neutral and Disease-Associated Human Genetic Variants through Interpretable Predictors
Source: PLoS One. 2015 Mar 31;10(3):e0120729. doi: 10.1371/journal.pone.0120729 (PMC4380319; doi:10.1371/journal.pone.0120729)
Supplement: S1 Table — The AAIndex amino acid scales with highest correlation (r) to the varimax-derived scales (V) as taken from [26]. (PDF) [file pone.0120729.s011.pdf]

Table S1: AAIndex scales with highest correlation (r) to the varimax-derived scales (V) as taken from [1].

V	**AAindex**	r	**Description**
1	NADH010102	0.969	Hydropathy scale based on self-information values in the two-state model (9% accessibility)
1	BIOV880101	0.968	Information value for accessibility; average fraction 35%
1	ROSG850102	0.965	Mean fractional area loss
2	PALJ810102	0.982	Normalized frequency of alpha-helix from CF
2	KANM800101	0.979	Average relative probability of helix
2	ISOY800101	0.978	Normalized relative frequency of alpha-helix
3	PONJ960101	0.893	Average volumes of residues
3	TSAJ990102	0.888	Volumes not including the crystallographic waters using the ProtOr
3	FAUJ880103	0.886	Normalized van der Waals volume
4	NAKH900101	0.954	AA composition of total proteins
4	JOND920101	0.954	Relative frequency of occurrence
4	CEDJ970102	0.953	Composition of amino acids in anchored proteins (percent)
5	BUNA790101	0.852	alpha-NH chemical shifts
5	FINA910102	-0.851	Helix initiation parameter at position $i, i+1, i+2$
5	AURR980119	-0.812	Normalized positional residue frequency at helix termini C“
6	AURR980117	0.811	Normalized positional residue frequency at helix termini C‘
6	FAUJ880107	-0.803	N.m.r. chemical shift of alpha-carbon
6	RACS820106	0.799	Average relative fractional occurrence in ER(i)
7	KLEP840101	0.932	Net charge
7	ZIMJ680104	0.875	Isoelectric
7	FINA910103	0.806	Helix termination parameter at position $j-2, j-1, j$
8	QIAN880117	0.753	Weights for beta-sheet at the window position of -3
8	QIAN880118	0.567	Weights for beta-sheet at the window position of -2
8	PALJ810110	0.556	Normalized frequency of beta-sheet in all-beta class
9	BUNA790103	0.646	Spin-spin coupling constants 3JHalpha-NH
9	JOND750102	-0.628	pK (-COOH)
9	FASG760105	-0.605	pK-C
10	MAXF760103	0.783	Normalized frequency of zeta R
10	DAYM780201	0.618	Relative mutability
10	WERD780102	0.607	Free energy change of epsilon(i) to epsilon(ex)

References

[1] Georgiev AG. Interpretable numerical descriptors of amino acid space. J Comput Biol. 2009;16(5):703–723.
