# Supplementary material for: Insight into Neutral and Disease-Associated Human Genetic Variants through Interpretable Predictors
Source: PLoS One. 2015 Mar 31;10(3):e0120729. doi: 10.1371/journal.pone.0120729 (PMC4380319; doi:10.1371/journal.pone.0120729)
Supplement: S2 Table — (PDF) [file pone.0120729.s012.pdf]

Table S2: Classifier performances

	all variants	per variant subset																	
		R	K	D	E	N	Q	S	G	H	T	A	P	V	M	C	L	I	WYF
C_S all features	0.833	0.792	0.777	0.819	0.793	0.831	0.797	0.827	0.827	0.832	0.816	0.830	0.809	0.827	0.825	0.821	0.871	0.833	0.790
C_E all features	0.813	0.780	0.746	0.797	0.778	0.808	0.801	0.817	0.785	0.828	0.802	0.821	0.794	0.798	0.810	0.767	0.844	0.785	0.766
PolyPhen2	0.807	0.765	0.756	0.800	0.770	0.841	0.791	0.810	0.810	0.835	0.784	0.808	0.793	0.794	0.845	0.808	0.783	0.823	0.784
SIFT	0.803	0.759	0.754	0.803	0.777	0.821	0.775	0.780	0.829	0.815	0.780	0.807	0.767	0.815	0.813	0.787	0.812	0.814	0.768
C_S AA subs	0.683	0.589	0.583	0.583	0.571	0.602	0.566	0.611	0.571	0.552	0.600	0.606	0.553	0.638	0.592	0.535	0.727	0.688	0.601
C_E AA subs	0.587	0.475	0.497	0.392	0.544	0.508	0.572	0.530	0.504	0.553	0.560	0.611	0.488	0.558	0.628	0.496	0.750	0.643	0.512
C_S sur. seq.	0.714	0.643	0.660	0.692	0.681	0.672	0.689	0.722	0.731	0.695	0.694	0.689	0.741	0.606	0.673	0.687	0.649	0.596	0.644
C_E sur. seq.	0.673	0.630	0.651	0.686	0.679	0.665	0.690	0.717	0.694	0.682	0.687	0.680	0.725	0.617	0.665	0.635	0.634	0.599	0.610
C_S cons.	0.775	0.746	0.703	0.787	0.748	0.795	0.729	0.763	0.740	0.779	0.742	0.772	0.766	0.742	0.767	0.756	0.779	0.767	0.733
C_E cons.	0.765	0.749	0.707	0.788	0.744	0.797	0.732	0.763	0.743	0.774	0.745	0.773	0.767	0.747	0.762	0.739	0.751	0.764	0.735
C_S phys. cons.	0.712	0.638	0.643	0.627	0.645	0.653	0.668	0.683	0.649	0.641	0.661	0.711	0.657	0.717	0.690	0.602	0.800	0.762	0.671
C_E phys. cons	0.633	0.599	0.467	0.573	0.543	0.558	0.657	0.648	0.542	0.584	0.587	0.668	0.629	0.707	0.614	0.561	0.768	0.567	0.548
C_S domain	0.720	0.651	0.662	0.646	0.666	0.650	0.647	0.686	0.626	0.673	0.678	0.680	0.671	0.652	0.664	0.632	0.657	0.638	0.626
C_E domain	0.676	0.670	0.700	0.675	0.687	0.681	0.671	0.701	0.642	0.706	0.705	0.701	0.682	0.671	0.694	0.653	0.684	0.672	0.649

AA subs: amino acid substitution, sur.seq.: surrounding sequence, cons.: conservation, phys. cons.: physicochemical conservation

C_S: combined subset classifiers, C_E: classifiers trained on entire set of variants
